# Persistence of a t(11;14)‐positive clone in a patient with mantle cell lymphoma for 20 years

**DOI:** 10.1002/ccr3.872

**Published:** 2017-03-02

**Authors:** Yasuyuki Otsuka, Momoko Nishikori, Toshiyuki Kitano, Tomomi Oka, Takayuki Ishikawa, Hironori Haga, Akifumi Takaori‐Kondo

**Affiliations:** ^1^ Department of Hematology and OncologyGraduate School of MedicineKyoto UniversityKyotoJapan; ^2^ Department of HematologyKobe City Medical Center General HospitalKobeJapan; ^3^ Department of PathologyKyoto University HospitalKyotoJapan

**Keywords:** Cyclin D1, mantle cell lymphoma, somatic hypermutation analysis, Sox11, Splenectomy

## Abstract

We report here a patient with extremely indolent mantle cell lymphoma (MCL) who had progressed and required immunochemotherapy 20 years after diagnostic splenectomy. Non‐nodal, indolent MCL patients may progress after such an extraordinary long indolent phase, and we recommend lifelong follow up for such cases.

Mantle cell lymphoma (MCL) is a distinct subtype of B‐cell neoplasms characterized by t(11;14)(q13;q32), which leads to constitutive deregulation of cyclin D1. Patients are typically diagnosed at over 60 years of age and have advanced‐stage disease. They are incurable with standard chemotherapy and exhibit an unfavorable clinical course with a median overall survival of 4–5 years. We report here a patient with splenic MCL who followed an extremely indolent clinical course. After long‐term remission following splenectomy, the patient finally progressed and received first immunochemotherapy 20 years after initial diagnosis.

A 48‐year‐old, previously healthy female was found to have an enlarged spleen without any symptoms and received diagnostic splenectomy in 1995. Histological examination revealed a monotonous proliferation of small‐to‐medium sized cleaved B cells in the white pulp, with weak CD5 and cyclin D1 expression. These B cells were shown to be positive for IgM*λ* by flow cytometry, and positive for t(11;14) by chromosomal analysis. According to these results, the patient was diagnosed with splenic MCL (Fig. 1A). Peripheral lymphocyte count was 4000/*μ*L and contained 20% of IgM*λ* B cells at presentation, but this cell population became undetectable after splenectomy. Because no other mass lesions were detected and only minimal involvement was found in the bone marrow, the patient was observed without additional treatment.

**Figure 1:**
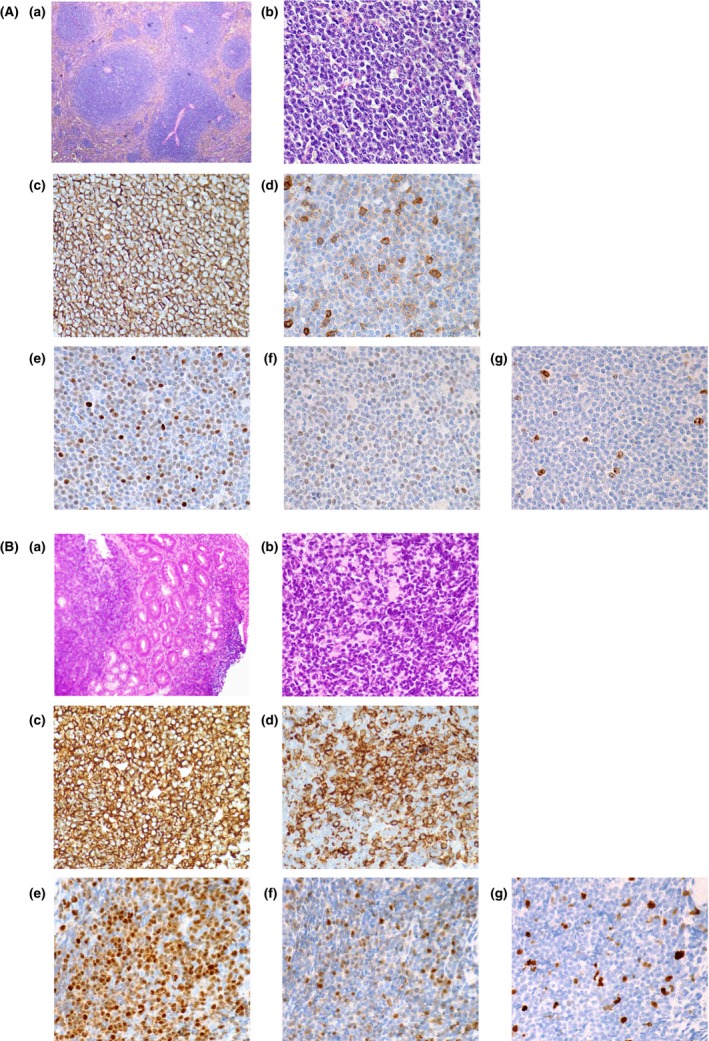
Histological images of lymphoma samples from the (A) spleen and (B) duodenum stained with (a, b) hematoxylin and eosin, (c) CD20 (L26; DAKO, Carpinteria, CA), (d) CD5 (4C7; Leica, Wetzler, Germany), (e) cyclin D1 (SP4‐R; Roche, Mannheim, Germany), (f) Sox11 (polyclonal, HPA000536; Sigma‐Aldrich, St Louis, MO), and (g) Ki‐67 (MIB‐1; DAKO). Original magnification, (a) ×40; (b–g), ×400.

She had long been asymptomatic, but developed dry cough after 18 years' remission following splenectomy. ^18^F‐fluoro‐2‐deoxy‐D‐glucose positron emission tomography and computed tomography (FDG‐PET/CT) scan found her lymphoma progressed at hilar lymph nodes, along with an increase in serum sIL‐2 receptor (Fig. 2A). Screening upper and lower endoscopic examination found slight elevation of the mucosa with a rough texture in the duodenum and ascending to transverse colon, and lymphoma involvement was confirmed by the biopsies of these lesions (Fig. 1B).

**Figure 2:**
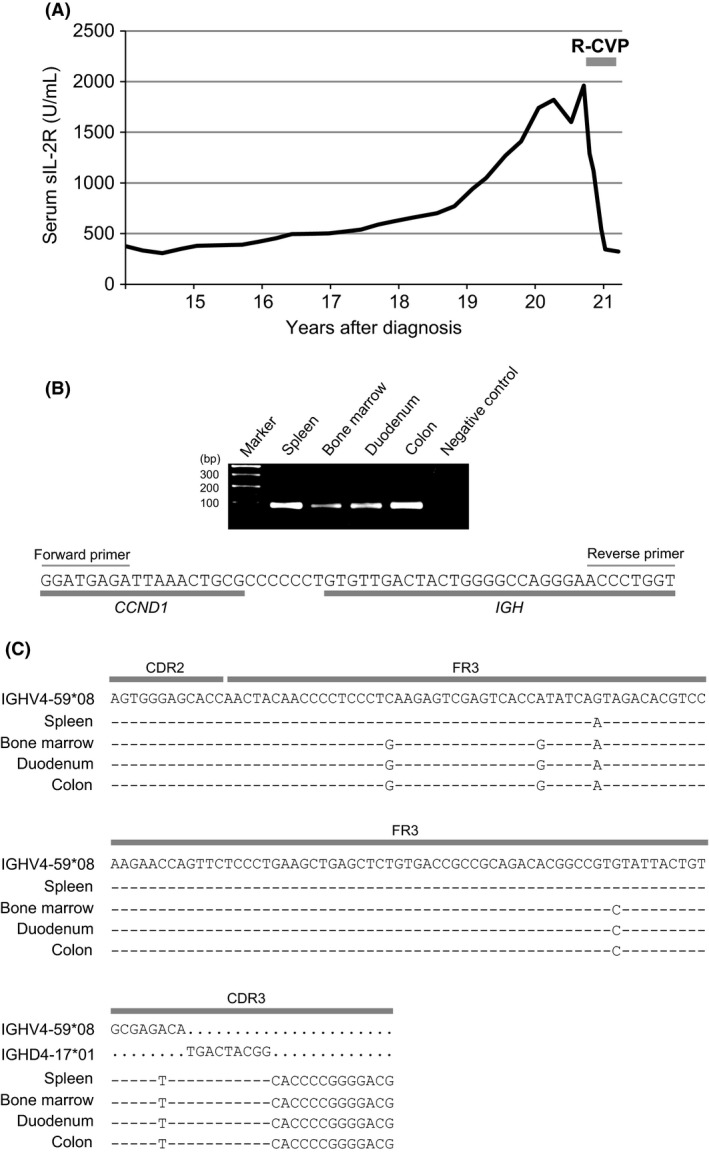
(A) Changes in serum sIL‐2R levels of the patient (normal range: 145–519 U/mL). (B) Results of PCR analysis for the detection of t(11;14) (forward primer: 5′‐CTCTTTATCTGAGTGGGATGAGA‐3′; reverse primer: 5′‐ACCTGAGGAGACGGTGACCAGGGT‐3′). As a negative control, genomic DNA from a healthy volunteer was used. Sequence alignment of the translocation break point junction is shown (common to all samples). (C) SHM analysis of the *IGH* variable region of the tumor cells. The tumor‐specific *IGH* variable region was PCR amplified (forward primer: 5′‐AGTGGGAGCACCAACTACAACCCCTCCC‐3′; reverse primer: 5′‐TAGTCAATCGTCCCCGGGGTGCCG‐3′), and SHM was analyzed by sequencing of the PCR products. CDR, complementarity‐determining region; FR, framework region.

Flow cytometry detected 5% CD5^+^ IgM*λ* ^+^ B cells in the bone marrow. Chromosomal analysis failed to detect the karyotype of the tumor cells, but fluorescence in situ hybridization (FISH) demonstrated that these tumor cells were positive for t(11;14) and negative for 17p deletion. Twenty years after splenectomy, she eventually received six cycles of R‐CVP therapy at the age of 68 and has been in complete remission for 7 months.

We examined the t(11;14) break point junctions by long‐distance polymerase chain reaction (PCR) 1 and Sanger sequencing of bone marrow‐derived genomic DNA samples extracted by the phenol–chloroform method. Then, we created tumor‐specific PCR primers for the detection of t(11;14) in DNA extracted from formalin‐fixed, paraffin‐embedded (FFPE) tumor samples using NucleoSpin^®^ DNA FFPE XS (Macherey‐Nagel, Duren, Germany). Sequence analysis of the PCR products showed that all the tumor cells from the spleen and those from the bone marrow, duodenum, and colon at progression carried the same t(11;14) junctional sequence (Fig. 2B), suggesting that they were clonally identical.

We next determined the tumor‐specific VDJ sequence of the *IGH* gene by semi‐nested PCR 2 using bone marrow‐derived DNA and created new PCR primers for the detection of the tumor‐specific VDJ sequence in FFPE‐extracted DNA samples. Somatic hypermutation (SHM) analysis of the PCR products found two mutations common to all samples and three additional mutations in the bone marrow and intestine samples, indicating that the tumor cells in the bone marrow and intestine had evolved from preceded splenic tumor cells (Fig. 2C). In accordance with these results, it was suggested that a minimal MCL clone remained in the patient following splenectomy, and it eventually evolved to systemic MCL and required treatment 20 years after the initial diagnosis.

The clinical course of MCL is usually aggressive, but a proportion of patients are recognized to have an indolent clinical course and do not require immediate treatment. In particular, a distinct subgroup of indolent MCL with a predominantly leukemic and splenic disease has been recognized 3. These non‐nodal, indolent MCL cells have several marked differences from conventional MCL cells, such as high SHM rate in the *IGH* gene and low expression of Sox11 4, 5. Palomero et al. have demonstrated that Sox11 promotes tumor angiogenesis through transcriptional regulation of PDGFA in MCL, and they hypothesized that non‐nodal localization of Sox11‐negative MCL cells may reflect their low angiogenic potential 6. They also suggested that Sox11 expression level separates MCL into two subtypes of different cell of origin because Sox11 has a function of repressing BCL6 transcription 7. Our patient originally had a typical non‐nodal MCL, and it eventually evolved into systemic MCL after a long period of time. Although the intestine is a frequently involved site in MCL patients, the intestinal tumors of the patient were atypical for MCL with weak Sox11 expression (Fig. 1B), which suggested their evolution from indolent MCL of a non‐nodal type. High SHM mutation rate in the *IGH* gene also supported their derivation from non‐nodal MCL.

Because the t(11;14) breakpoint junctions are found in the variable region of the *IGH* gene, t(11;14) translocation is considered to be generated by an error during VDJ rearrangement in the early B‐cell developmental stage, just the same as the t(14;18) translocation. It has been suggested that t(11;14)‐positive clonal B cells can be detected in healthy individuals at very low levels, and only a minority of them subsequently develop into MCL. However, the natural history of t(11;14)‐positive B cells is only marginally recognized, in contrast to t(14;18)‐positive B cells, which has accumulated evidence for their long‐term clonal persistence. This may be due to the lower frequency of t(11;14)‐positive B cells in healthy individuals compared with t(14;18)‐positive B cells, and the poor outcome of MCL, which prevents the opportunity for long‐term observation.

Mantle cell lymphoma cells of a non‐nodal type are reported to carry very few chromosomal alterations compared with conventional systemic MCL 5. Therefore, it can be speculated that the primary splenic tumor cells of our patient required decades to acquire additional genetic abnormalities to evolve into systemic MCL, at the typical age range for MCL. To the best of our knowledge, this is the only report demonstrating this length of persistence of a t(11;14)‐positive clone in a MCL patient during remission. Our report adds an important evidence that non‐nodal MCL patients may progress after such an extraordinary long indolent phase. According to our experience, we recommend lifelong follow up for these patients.

## Authorship

YO: performed genetic analysis; MN: drafted the manuscript; MN, TK, TO and TI: participated in the clinical care of the patient; HH: performed histological diagnosis: and ATK: supervised the manuscript.

## Conflicts of Interest

The authors have no financial conflict of interests to disclose with regard to this report.
